# A Novel Nude Mice Model for Studying the Pathogenesis of Keloid

**DOI:** 10.1111/jocd.70284

**Published:** 2025-06-09

**Authors:** Ying Zhang, Yanqiu Bao, Zhouna Li, Zhehu Jin

**Affiliations:** ^1^ Department of Dermatology Shaoxing People's Hospital (Shaoxing Hospital, Zhejiang University School of Medicine) Shaoxing Zhejiang China; ^2^ Department of Dermatology Affiliated Hospital of Yanbian University Yanji Jilin China; ^3^ Department of Medical Cosmetology and Plastic Surgery Shandong Maternal and Child Health Care Hospital Jinan Shandong China

**Keywords:** fibroblast, keloid, matrigel, model, nude mice

## Abstract

**Background:**

Keloid is a common fibroproliferative disorder, often manifesting symptoms such as itching and pain. Given the unique characteristics of keloids, their occurrence is primarily limited to humans, posing difficulties for spontaneous keloid development in animals. Consequently, the creation of animal models has somewhat impeded the comprehensive investigation of keloids.

**Aims:**

The aim is to develop a keloid mouse model that mimics the formation process of human keloids, enabling researchers to gain a deeper understanding of their pathophysiological mechanisms and to explore effective therapeutic approaches.

**Methods:**

Human keloid fibroblasts were isolated, cultured, and subcutaneously injected with Matrigel into nude mice at four concentrations (1 × 10^6^, 3 × 10^6^, 5 × 10^6^, and 7 × 10^6^ cells). Subcutaneous nodules were transplanted into additional mice to validate stability. Histological and immunohistochemical analyses were performed to assess morphological and molecular features.

**Results:**

Keloid‐like nodules formed in a cell density‐dependent manner, with the highest concentration group (7 × 10^6^ cells) achieving nodule formation in 39.6 ± 3.2 days, significantly faster than lower concentrations (*p* < 0.001). The transplantation success rate reached 80%, with nodules exhibiting dense collagen deposition (72.7% ± 3.8%), CD34‐positive microvessels (23.1 ± 2.2 vessels/field), and α‐SMA expression (11.29% ± 3.7%), closely mirroring human keloid histopathology.

**Conclusion:**

The combination of human keloid fibroblasts and Matrigel provides a simple and rational approach for constructing a nude mouse model, offering a reliable animal model for experimental and clinical research on the pathogenesis and treatment strategies of keloids.

## 1 Introduction

Keloid is a chronic dermatological condition distinguished by aberrant fibroblast proliferation and excessive deposition of collagen [1]. Although considered a benign proliferative disorder, keloid substantially affects the quality of life, physical health, and psychological well‐being of patients [2]. Despite the existence of diverse treatment approaches, including surgical excision, radiotherapy, laser therapy, and intralesional drug injection, the therapeutic outcomes for keloid often fall short of optimal, and preventing recurrence remains a formidable challenge. The identification of an efficacious treatment strategy remains an unresolved clinical matter. Given the distinct characteristics of keloid disease, primarily observed in the human body, the establishment of a reliable and consistent animal model is an essential prerequisite for studying the pathogenesis and therapeutic interventions for keloid.

Numerous experimental endeavors have been undertaken to establish animal models of spontaneous keloid in mice, rabbits, and pigs. Nevertheless, the absence of keloid formation in these animals may be attributed to dissimilarities in skin physiology, wound healing patterns, and other pertinent factors when compared to humans [3]. Conversely, horses manifest a proliferative scarring phenotype that bears resemblance to human keloid [4]. Nonetheless, the utilization of these models is constrained by their exorbitant expenses and protracted cycles, thereby rendering them unfeasible for extensive experimentation. Rodents are frequently employed as experimental animals; however, the occurrence of spontaneous keloid formation in rodents has not been documented to date. As a result, the prevailing animal models for keloid research involve the surgical implantation of human keloid tissue beneath the skin of immunodeficient mice. These models are influenced by various factors, including the availability of excised scar tissue, individual differences in the source of keloid tissue, and limitations on the subcutaneous placement of the implants [3]. Consequently, the exploration of novel animal models is imperative to further the advancement of keloid disease research.

The primary objective of this study was to develop a keloid animal model by employing a combination of human keloid fibroblasts and Matrigel. The aim was to construct a simplified and rational nude mouse model that accurately reflects the biological attributes of keloid. This model is intended to serve as an ideal animal model for both experimental and clinical investigations pertaining to keloid.

## 2 Methods and Materials

### 2.1 Experimental Animals

Fifty‐five female BALB/c nude mice, aged approximately 7 weeks and weighing between 17 and 19 g, were procured from Changchun Yisi Experimental Animal Technology Co. Ltd. (Changchun, China). The mice were housed in a specific pathogen‐free environment, with a room temperature maintained at 20°C–25°C and humidity levels between 40% and 70%. The nude mice were subjected to a 12/12‐h light/dark cycle and provided with unrestricted access to sterile drinking water and feed.

### 2.2 Isolation and Cultivation of Human Keloid Fibroblasts

Skin tissues obtained from patients diagnosed with keloids were collected and subsequently washed with a phosphate‐buffered saline (PBS, Gibco, C10010500BT) solution supplemented with penicillin and streptomycin. These fragments were then carefully placed in 60 mm culture dishes and subjected to enzymatic digestion using 3 mg/mL collagenase I and 4 mg/mL digestion enzyme. The digestion process was carried out at a temperature of 37°C for a duration of 1 h. After digestion, the cells underwent centrifugation to obtain a cell pellet, which was subsequently washed twice with Dulbecco's Modified Eagle Media (Gibco, 11 885 084) and seeded in 60 mm culture dishes, labeled as primary cells (P0). Once the cells reached 80% confluence, enzymatic dissociation was performed, followed by filtration through a 70 μm cell strainer. Subsequently, 3 × 10^6^ cells/mL were seeded in 10 mm culture dishes for further cultivation, referred to as the first passage (P1). Upon reaching 90% confluence, the cells were passaged at a 1:4 ratio into new culture dishes. By the third passage, the cells were deemed suitable for subsequent experimental use. Human keloid tissues were obtained with informed consent from patients, and the study protocol was approved by the Ethics Committee of the Affiliated Hospital of Yanbian University (Yan‐Yi Ethics 2 016 017).

### 2.3 Injecting Human Keloid Fibroblasts Into Nude Mice

**FIGURE 1:**
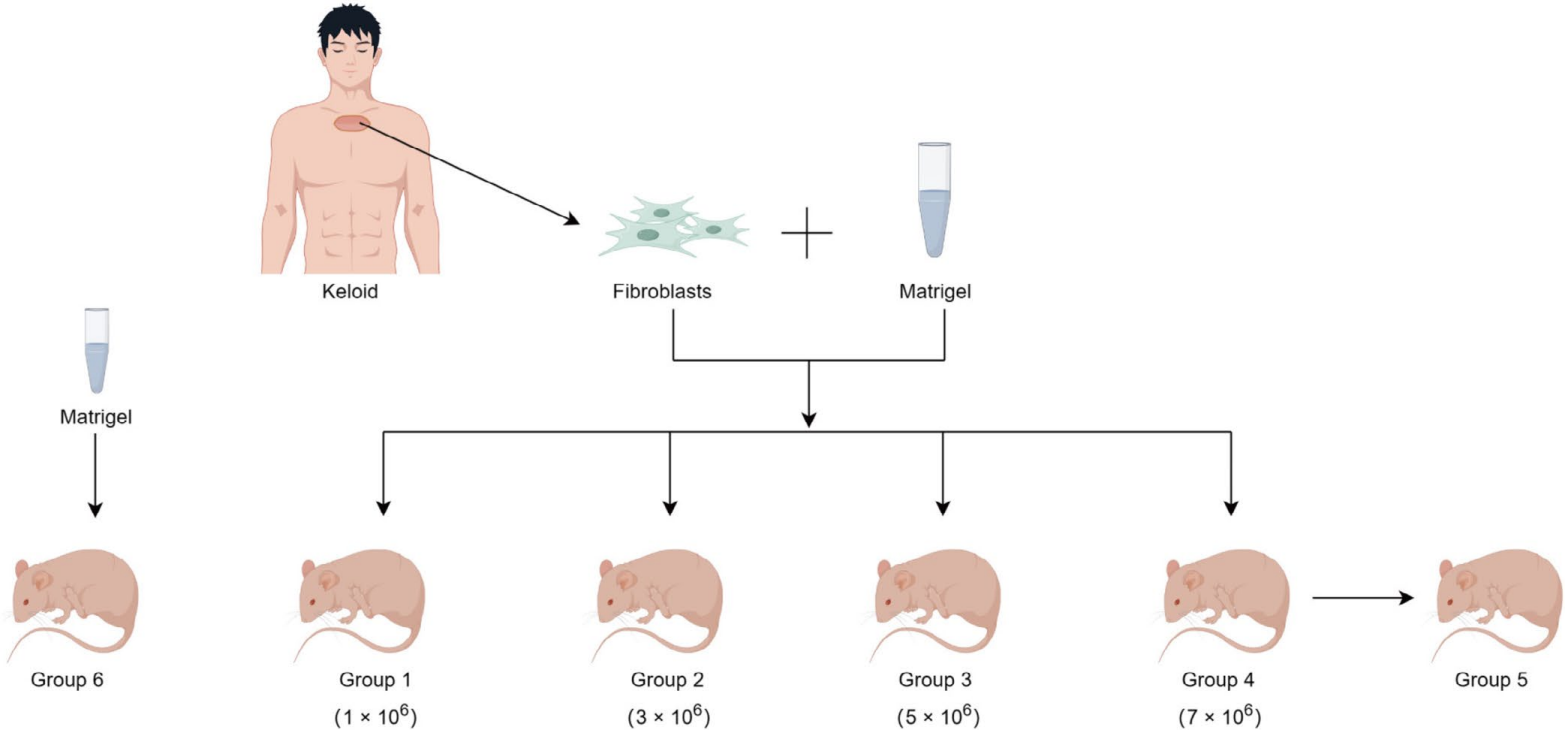
Experimental grouping workflow diagram.

A total of 55 nude mice were subjected to random allocation into six distinct groups for the purpose of this study. Specifically, Group 1 consisted of eight mice that were injected with 1 × 10^6^ cells, while Group 2 comprised eight mice that received an injection of 3 × 10^6^ cells. Similarly, Group 3 included eight mice that were injected with 5 × 10^6^ cells, and Group 4 consisted of eight mice that received an injection of 7 × 10^6^ cells. Additionally, Group 5 was designated for tumor transplantation and consisted of 15 mice. Lastly, Group 6, serving as a control, comprised eight mice that were injected with 100 μL of Matrigel (Corning, 356 234). To generate a 100 μL volume of the mixture, each cell group was resuspended in PBS and subsequently combined with Matrigel at a 1:1 ratio. The detailed subgrouping is depicted in Figure 1, by Figdraw (https://www.figdraw.com/static/index.html#/paint_index_v2).

### 2.4 Nude Mouse Keloid Tissue Transplantation Experiment

When the formation of keloid‐like lesions was observed in the right axilla of nude mice injected with human keloid fibroblasts, euthanasia was performed. Following this, the subcutaneous keloid‐like lesions were excised and reduced to a volume of 2 × 2 × 2 mm. A longitudinal incision measuring approximately 0.3 cm was made in the right axilla of the fifth group of nude mice, and the subcutaneous tissues were carefully and gently dissected without causing trauma. The trimmed tissues were subsequently implanted subcutaneously, and the incision was closed using 5–0 sutures.

### 2.5 Histopathological Analysis of Keloid Lesions in Nude Mice

The excised tissues were submerged in a 10% formalin solution, subsequently embedded in paraffin, and sliced into sections measuring 4 μm in thickness. These tissue sections were subjected to staining with hematoxylin and eosin (H&E), Masson's trichrome staining, Sirius Red staining, as well as immunohistochemical (IHC) staining. The CD34 antibody, obtained from Santa Cruz, was diluted at a ratio of 1:500. The α‐SMA antibody obtained from Proteintech (Wuhan, China) was diluted at a ratio of 1:1000. Use an optical microscope to capture images of H&E staining, Masson staining, and IHC staining. The results of Sirius Red staining were observed with a polarized light microscope. Stained images were quantified using ImageJ software.

### 2.6 Statistical Analysis

The statistical analysis was conducted using SPSS 26.0 software. Continuous data were presented as mean ± SD. Between‐group comparisons were performed using analysis of variance, with a significance level of *p* < 0.05.

## 3 Results

### 3.1 Fibroblasts From Human Keloids at Various Concentrations Are All Applicable for Establishing Keloid‐Like Lesions in Nude Mice

**FIGURE 2:**
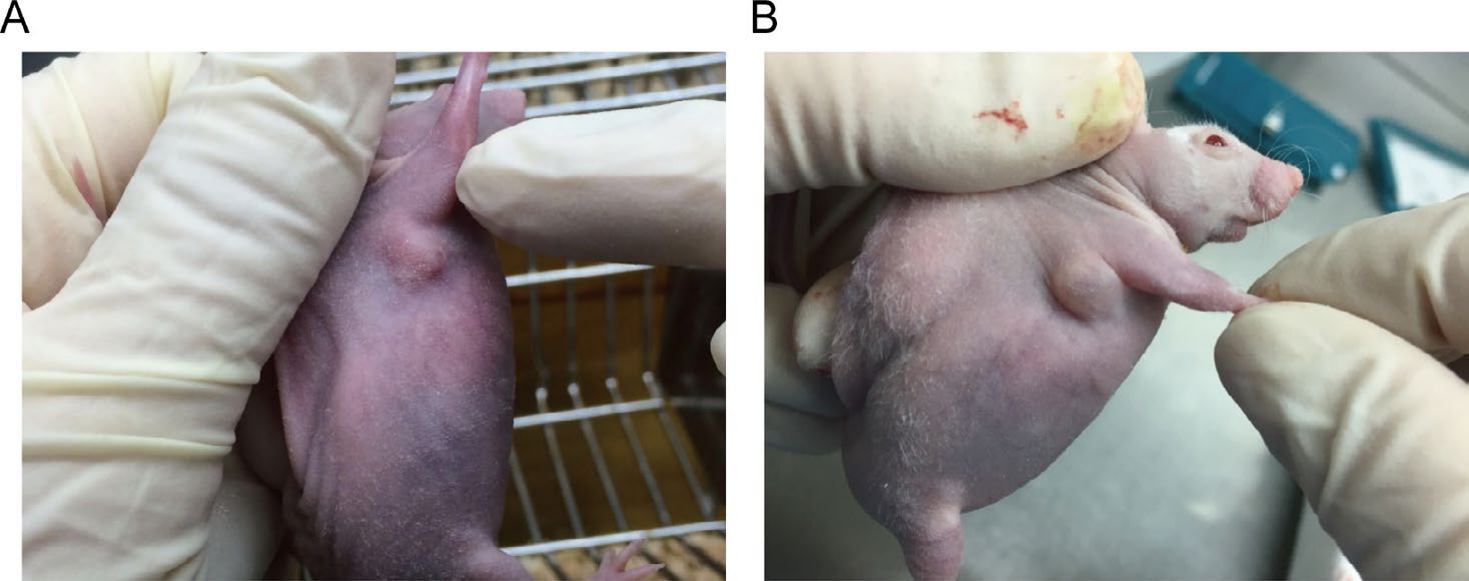
Subcutaneous nodule growth following inoculation of fibroblasts derived from human keloid tissues. (A) Group 1 nude mice, with subcutaneous injection of 1 × 10^6^ cells. (B) Group 4 nude mice, with subcutaneous injection of 7 × 10^6^ cells.

**FIGURE 3:**
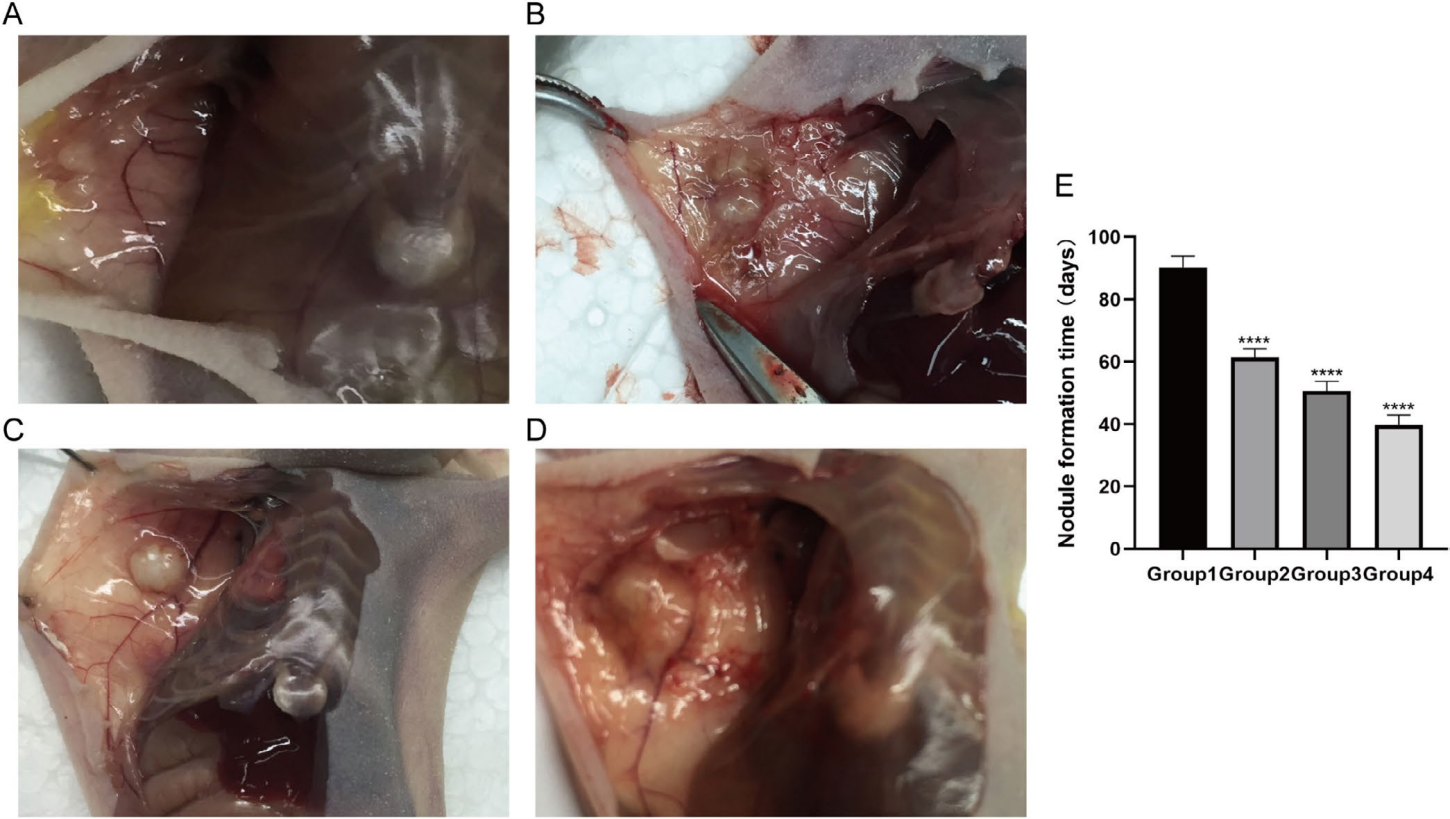
Growth patterns of subcutaneous nodules in nude mice following inoculation with different concentrations of human keloid‐derived fibroblasts. (A) Group 1 nude mice, with subcutaneous injection of 1 × 10^6^ cells. (B) Group 2 nude mice, with subcutaneous injection of 3 × 10^6^ cells. (C) Group 3 nude mice, with subcutaneous injection of 5 × 10^6^ cells. (D) Group 4 nude mice, with subcutaneous injection of 7 × 10^6^ cells. (E) Growth rate analysis across varying cell concentrations. **** is statiscally different methods used for t‐tests.

In order to evaluate the influence of human keloid fibroblast concentration on the development of keloid‐like lesions in nude mice, this study conducted subcutaneous implantation of 1 × 10^6^, 3 × 10^6^, 5 × 10^6^, and 7 × 10^6^ cells into the nude mice. The findings of the experiment demonstrate that nude mice implanted with varying cell concentrations exhibited the formation of subcutaneous nodules. The time at which the nodules reached a diameter of ≥ 5 mm was recorded as the point of nodule formation. The time required for nodule formation in each experimental group was recorded as follows: Group 1 (90.2 ± 2.2) days, Group 2 (61.3 ± 2.9) days, Group 3 (50.6 ± 3.2) days, and Group 4 (39.6 ± 3.2) days. Subsequent observation for an additional 3 weeks after nodule formation revealed a discernible trend: the higher the concentration of implanted cells, the more rapid the growth of subcutaneous nodules (Figure 2). Upon dissection, it was noted that the subcutaneous nodules in Group 4 of nude mice exhibited the greatest volume (Figure 3).

### 3.2 Subcutaneous Transplantation to Establish a Keloid Nude Mouse Model

**FIGURE 4:**
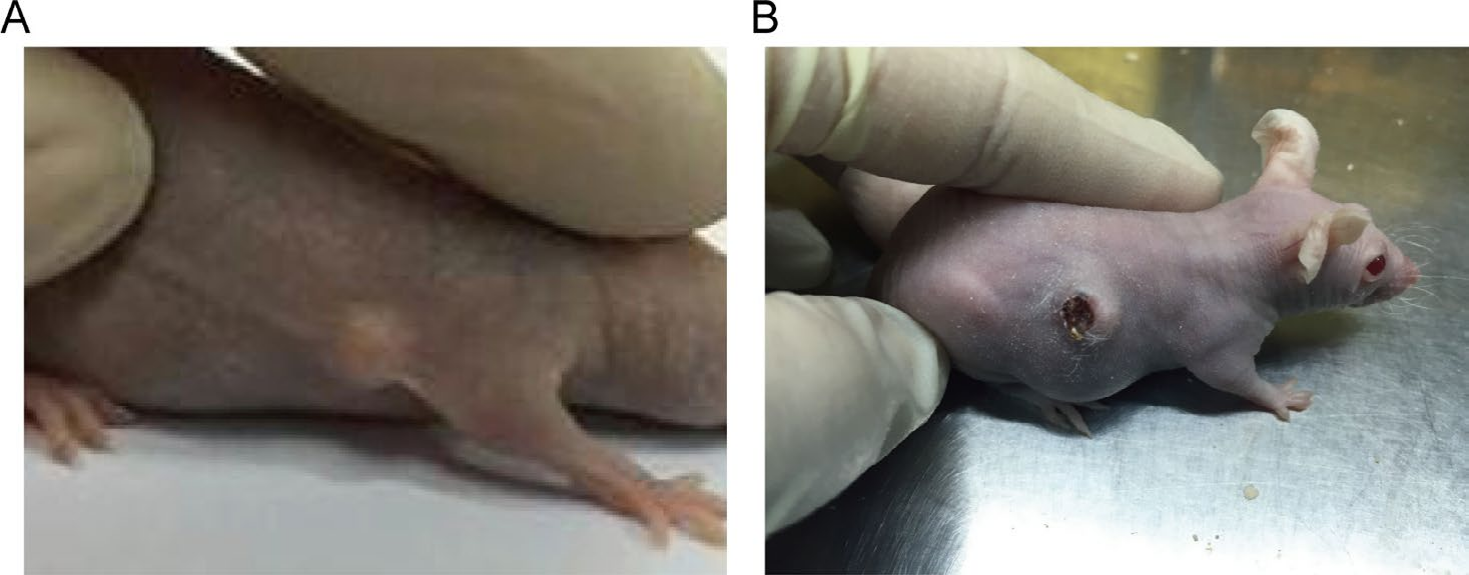
Growth status of subcutaneous nodules after transplantation. (A) Group 5 nude mice with successful subcutaneous tissue transplantation. (B) Group 5 nude mice exhibiting scab formation at the transplant site, indicating unsuccessful transplantation.

The subcutaneous nodule tissue obtained from Group 4 nude mice was surgically removed, trimmed to a standardized volume of 2 × 2 × 2 mm, and subsequently transplanted subcutaneously into Group 5 nude mice. Analysis of the experimental data indicated that, during the early phase following transplantation, the volume of subcutaneous nodules exhibited a slight initial increase, followed by a gradual decrease after 4 days, and eventually displayed a progressive increase around the 2‐week mark. During the experimental process, mortality was observed in one nude mouse, and two nude mice exhibited incisional breakdown at the transplantation site with no subcutaneous nodule formation (Figure 4). Consequently, in Group 5, a cumulative total of 13 nude mice were effectively transplanted, resulting in a keloid formation rate of 80%.

### 3.3 Histological Analysis of Keloid Lesions in Nude Mice

**FIGURE 5:**
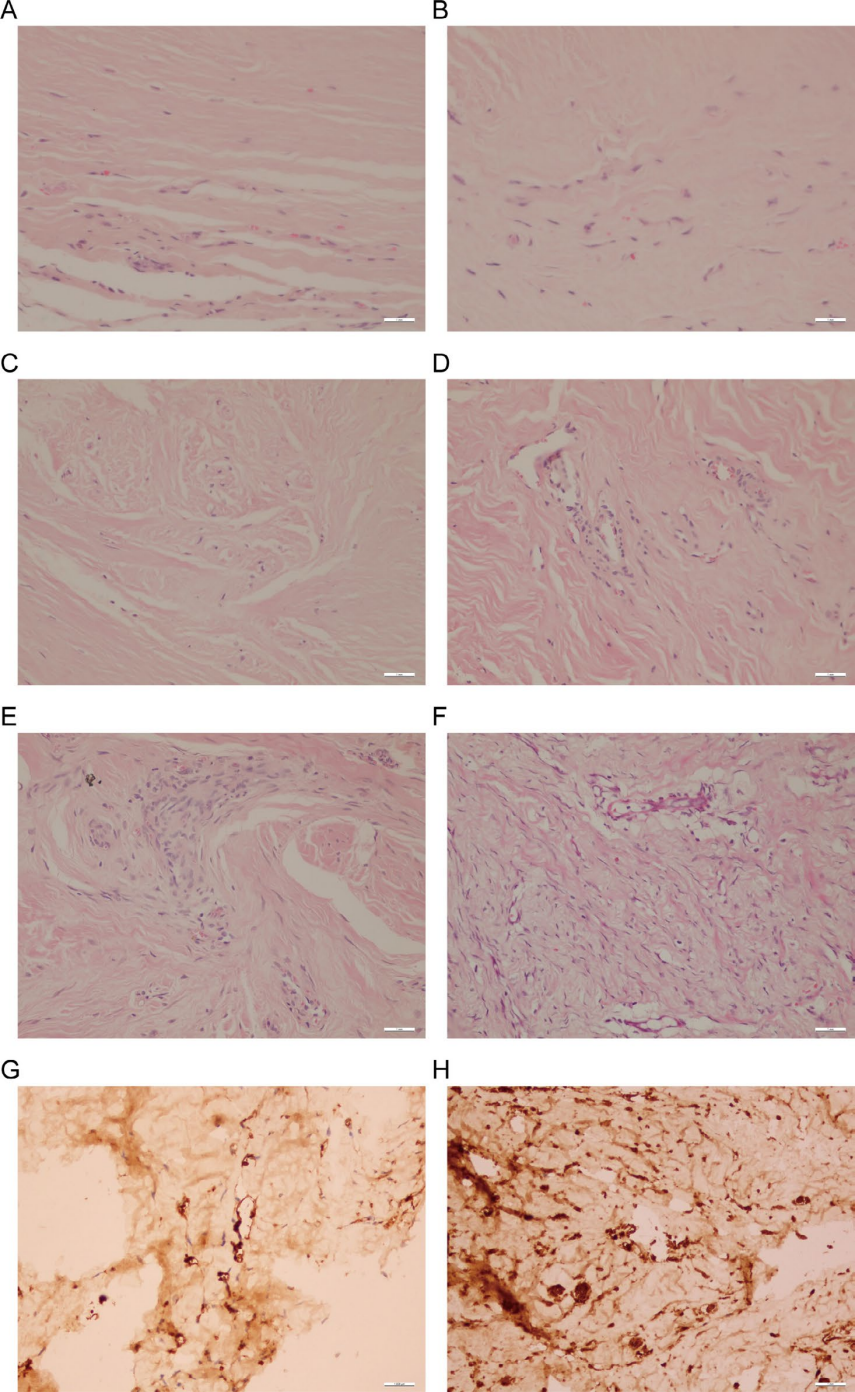
Histological morphology of subcutaneous nodules in nude mice and human keloid tissues. (A–E) H&E stained images of subcutaneous tissues from Group 1 to Group 5 nude mice, 20×. (F) H&E stained image of human keloid tissue, 20×. (G) Immunohistochemical images of CD34 expression in newly formed subcutaneous tissue of Group 4 nude mice, 40×. (H) Immunohistochemical images of CD34 expression in newly formed subcutaneous tissue of Group 5 nude mice, 20×.

**FIGURE 6:**
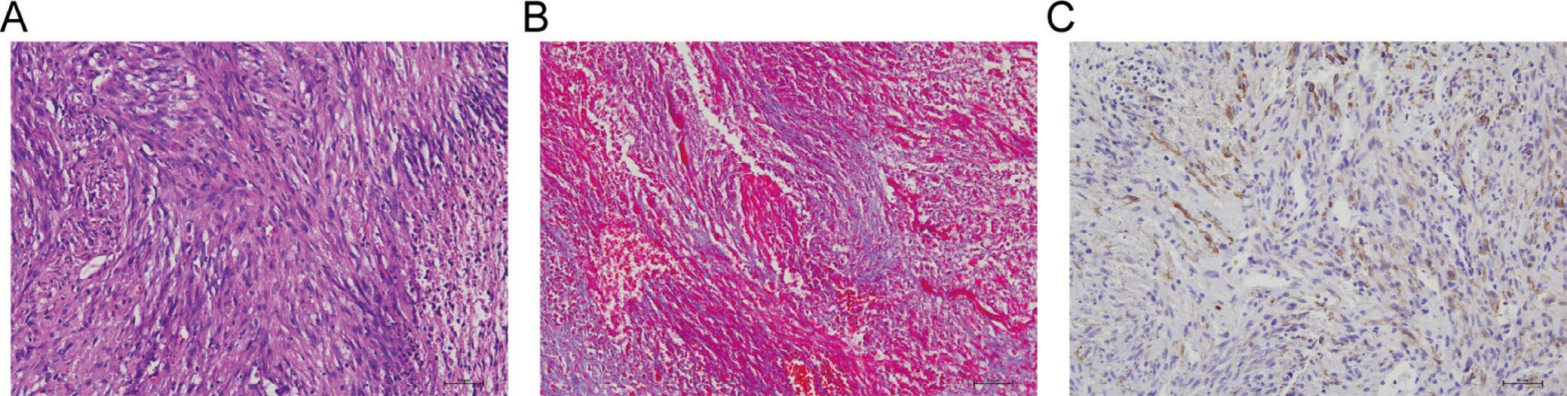
Various staining techniques were used to assess the collagen fiber composition in keloid‐like tissues. (A) H&E staining of keloid‐like tissue in nude mice, 20×. (B) Masson's trichrome staining of keloid‐like tissue, 20×. (C) Immunohistochemical staining of α‐SMA in keloid‐like tissue, 20 ×.

**FIGURE 7:**
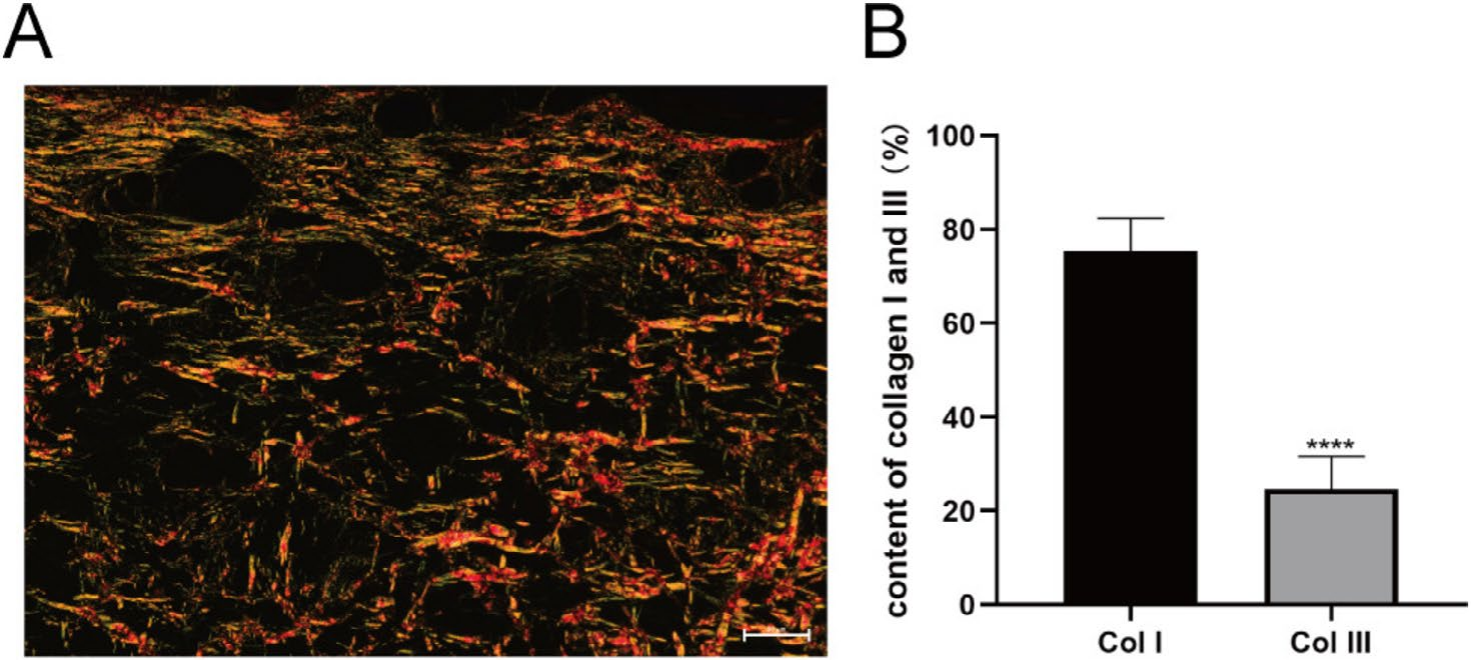
(A) Sirius red staining of keloid‐like tissue, where yellow regions indicate type I collagen and green regions indicate type III collagen, 20×. (B) Quantitative analysis of collagen. **** is statiscally different methods used for t‐tests.

The subcutaneous nodules observed in nude mice from Groups 1 to 5 exhibited morphological characteristics congruent with the microscopic structure of human keloid tissues, including densely packed fibroblasts with disorganized arrangements (Figure 5A–F). Immunohistochemical analysis revealed robust CD34‐positive microvessels in Group 5 (23.1 ± 2.2 vessels/field), reflecting active angiogenesis akin to human keloids. Masson's trichrome staining demonstrated elevated collagen deposition in nodules (72.7% ± 3.8% collagen density, Figure 6B), while α‐SMA immunohistochemistry confirmed myofibroblast activity (11.29% ± 3.7% positive area, Figure 6C). Sirius Red staining further quantified collagen composition, showing 75.4% ± 7.0% type I collagen in experimental groups (Figure 7), closely resembling human keloid collagen profiles. These combined analyses validate the model's fidelity to human keloid pathology.

## 4 Discussion

Keloid, a chronic fibroproliferative disorder, exerts substantial effects on the physical and mental well‐being of those affected. The intricate pathogenesis of this condition presents a formidable obstacle for clinicians and researchers in their pursuit of effective treatments [5, 6]. Moreover, the spontaneous formation of keloid lesions in animal models poses a significant challenge, given the unique human‐specific nature of this pathology. Consequently, the development of suitable animal models becomes imperative for comprehensively investigating the pathogenesis and therapeutic interventions for keloid.

Presently, there exist two overarching classifications for common animal models utilized in keloid research. The first classification pertains to the keloid implantation model, wherein human keloid tissue is subcutaneously transplanted into immunodeficient mice. Preliminary investigations have demonstrated that this model can be maintained for a minimum of 60 days without eliciting any rejection responses [7]. Further studies have indicated a reduction in the weight of the transplanted tissue after 80 days, thereby indicating the suitability of this model for short‐term experimental inquiries [8]. Nevertheless, it is important to acknowledge that this method of model preparation possesses certain limitations [3]. The transplantation of human keloid tissue into nude mice should be performed within a time frame of 2–3 h after excision to prevent necrosis and ensure successful modeling. It is important to note that the keloid tissue used for transplantation is obtained from a limited portion of the original tissue, potentially affecting the representation of the entire pathological structure and subsequent experimental results. The presence of diverse sources of human keloid tissue, which are influenced by various factors such as patient age and gender, poses a significant challenge in maintaining consistency within the nude mouse keloid model. An alternative approach involves employing a tissue engineering model, wherein keloid‐derived cells are utilized to engineer tissues that can be transplanted into mice for in situ or in vitro studies. Wang et al. successfully isolated fibroblasts from human keloid tissue, incorporated them into a polylactic‐co‐glycolic acid copolymer to create an in vitro composite, and subsequently transplanted this composite subcutaneously into nude mice to establish a keloid model [9]. However, the utilization of this approach necessitates stringent criteria for biomaterials, culture systems, and operational techniques, thereby impeding its widespread application in keloid research.

To evaluate the influence of varying concentrations of human keloid fibroblasts on the development of nude mouse models, we established four cell concentration gradients based on preliminary experimental results: 1 × 10^6^, 3 × 10^6^, 5 × 10^6^, and 7 × 10^6^ cells. Our experimental findings demonstrated a noteworthy association between cell density and the duration required for subcutaneous nodule formation. Notably, when the seeding density reached 7 × 10^6^ cells, the time for nodule formation was approximately 40 days, suggesting it to be a relatively suitable inoculation density. However, this requires the cultivation of a significant number of human keloid fibroblasts. Consequently, in this study, pre‐existing keloid tissue was trimmed and subsequently transplanted subcutaneously into additional nude mice. During the initial stages following transplantation, there was a slight increase in the volume of the subcutaneous nodule, which then gradually decreased. This occurrence can potentially be attributed to edema induced by trauma in the subcutaneous tissue during the transplantation process, leading to a minor volume increase. Visual observation revealed that the subcutaneous nodules were solid, round, or oval in shape, with newly formed blood vessels visible at the growth sites. The nodules had intact capsules and showed no invasion of surrounding tissues. The cross‐sections of the nodules were pale red and had a soft texture. Microscopic examination revealed significant inflammatory cell infiltration, a markedly increased density of fibroblasts, and disorganized cell arrangement. Collagen fibers were densely packed and irregularly arranged, with newly formed microvessels observed in some regions. These features closely resemble the pathological characteristics of human keloid tissue, further confirming the reliability of the keloid nude mouse model we established. These findings provide evidence supporting the stability and consistency of the experimental model. Table 1 shows the differences between our model and previous methods.

**TABLE 1:** Comparison of keloid animal models.

Model type	Advantages	Limitations
Human tissue transplant	Direct use of human keloid tissue	Dependent on donor tissue; high variability
Tissue‐engineered	Customizable scaffold‐cell interactions	Technically complex; high material requirements
Our model	Standardized cell density; rapid nodule formation (40 days); high reproducibility	Matrigel components may influence outcomes

Previous research has employed human keloid fibroblasts to establish keloid nude mouse models [10]. Compared to previous approaches using human keloid fibroblasts, we simulated the modeling methods of tumor animal models. Matrigel is a commonly used auxiliary material for preparing animal models of tumors. It remains in a liquid state at 4°C and polymerizes to form a biologically active three‐dimensional matrix at room temperature, which can mimic the structure of the cell basement membrane in vivo. When mixed with tumor cells, this substance can shorten the time required for tumor formation and increase the rate of tumor formation. By utilizing this characteristic of Matrigel, we mixed it with human fibroblasts and transplanted the mixture subcutaneously into nude mice to create a keloid model. Additionally, we explored the effects of different cell inoculation densities. Our study found that the most appropriate cell seeding concentration is 7 × 10^6^ cells. However, further research is needed to investigate the impact of cell passage number and cell viability on the construction of the animal model.

However, this method also has certain limitations. Matrigel contains type IV collagen, metalloproteinases, and growth factors, which may have potential impacts on the experimental results. Moreover, the use of immunodeficient animals in this study to prevent human cell rejection precludes the study of the immune system's role in keloid formation [11]. To address this limitation, we included a control group in our experimental design, in which only Matrigel was injected, to evaluate its impact on the experimental outcomes. Nevertheless, efforts should be made in future applications to minimize the use of Matrigel or select more suitable alternative materials to reduce its potential impact on the results. Future research could focus on developing “humanized” mice that incorporate components of the human immune system, to more comprehensively study the immune system's role in keloid formation [12].

In summary, the utilization of human keloid fibroblasts in conjunction with Matrigel has proven effective in inducing keloid lesions in nude mice. This experimental model not only facilitates the study of therapeutic approaches for keloids but also holds significant value in investigating gene modifications or abnormal protein expressions in the pathogenesis of keloids.

## Author Contributions

Drafting the manuscript: Y.Z. and Y.B. Model investigation and analysis: Y.Z., Y.B. and Z.L. Molecular Biology and Pathology Experiments: Y.Z., Y.B., Z.L., and Z.J. Data curation: Z.L. Conception and design, methodology, resources, writing – review and editing: Y.Z., Y.B., Z.L., and Z.J. All authors disclosed no relevant relationships.

## Ethics Statement

The authors confirm that the ethical policies of the journal, as noted on the journal's author guidelines page, have been adhered to and the appropriate ethical review committee approval has been received. The US National Research Council's guidelines for the Care and Use of Laboratory Animals were followed. This study obtained ethical approval from the Medical Ethics Committee of Yanbian University Affiliated Hospital, with the assigned ethics approval number: Yan‐Yi Ethics 2016017.

## Conflicts of Interest

The authors declare no conflicts of interest.

## Data Availability

The data that support the findings of this study are available from the corresponding authors upon reasonable request.
